# 胸腺切除范围对早期胸腺上皮肿瘤预后的影响

**DOI:** 10.3779/j.issn.1009-3419.2016.07.08

**Published:** 2016-07-20

**Authors:** 志涛 谷, 剑华 傅, 毅 沈, 煜程 魏, 黎杰 谭, 鹏 张, 泳涛 韩, 椿 陈, 仁泉 张, 印 李, 克能 陈, 和忠 陈, 永煜 刘, 有斌 崔, 允 王, 烈文 庞, 振涛 于, 鑫明 周, 阳春 柳, 媛 刘, 文涛 方, 中国胸腺肿瘤协作组成员

**Affiliations:** 1 200030 上海，上海交通大学附属上海胸科医院 Department of Thoracic Surgery, Shanghai Chest Hospital, Shanghai Jiao Tong University, Shanghai 200030, China; 2 510060 广州，中山大学附属肿瘤医院胸外科 Department of Thoracic Surgery, Guangdong Esophageal Cancer Institute, Sun Yat-sen University Cancer Center, State Key Laboratory of Oncology in South China, Collaborative Innovation Center of Cancer Medicine, Guangzhou 510060, China; 3 266001 青岛大学医学院附属医院胸外科 Department of Thoracic Surgery, Afliated Hospital of Qingdao University, Qingdao 266001, China; 4 200032 上海，复旦大学附属中山医院胸外科 Department of Thoracic Surgery, Zhongshan Hospital, Fudan University, Shanghai 200032, China; 5 300052 天津，天津 医科大学附属总医院胸外科 Department of Endocrinology, Tianjin Medical University General Hospital, Tianjin 300052, China; 6 610041 成都，四川省肿瘤医院胸外科 Department of Thoracic Surgery, Sichuan Cancer Hospital, Chengdu 610041, China; 7 350001 福州，福建医科大学附属协和医院胸外 科 Department of Thoracic Surgery, Fujian Medical University Union Hospital, Fuzhou 350001, China; 8 230022 合肥，安徽医科大学附属第一医院胸外科 Department of Thoracic Surgery, First Afliated Hospital of Anhui Medical University, Hefei 230022, China; 9 450008 郑州，郑州大学附属肿瘤医院胸外科 Department of Thoracic Surgery, Afliated Cancer Hospital of Zhengzhou University, Zhengzhou 450008, China; 10 100142 北 京，北京大学附肿瘤医院胸外科 Department of Thoracic Surgery, Beijing Cancer Hospital, Beijing 100142, China; 11 200433 上海，长海医院胸心外科 Department of Cardiothoracic Surgery, Changhai Hospital, Shanghai 200433, China; 12 110042 沈阳，辽宁肿瘤医院胸外科 Department of Thoracic Surgery, Liaoning Cancer Hospital, Shenyang 110042, China; 13 130021 长春，吉林大学附属第一医院胸外科 Department of Thoracic Surgery, First Afliated Hospital of Jilin University, Changchun 130021, China; 14 610041 成都，四川大学华西医院胸外科 Department of Thoracic Surgery, West China Hospital, Sichuan University, Chengdu 610041, China; 15 200032 上海，复旦大学附属华山 医院胸外科 Department of Thoracic Surgery, Huashan Hospital, Fudan University, Shanghai 200032, China; 16 300060 天津，天津医科大学附属肿瘤医院食管癌中心 Department of Esophageal Cancer, Tianjin Cancer Hospital, Tianjin 300060, China; 17 310022 杭州，浙江省肿瘤医院胸外科 Department of Thoracic Surgery, Zhejiang Cancer Hospital, Hangzhou 310022, China; 18 330006 南昌，江西省人民医院胸外科 Department of Thoracic Surgery, Jiangxi People's Hospital, Nanchang 330006, China

**Keywords:** 胸腺上皮肿瘤, 重症肌无力, 胸腺切除, 胸腺瘤切除, Thymic epithelial tumors, Myasthenia gravis, Tymectomy, Tymomectomy

## Abstract

**背景与目的:**

探采用中国胸腺肿瘤协作组胸腺肿瘤多中心回顾性数据库，探讨胸腺切除范围对早期胸腺上皮肿瘤预后的影响。

**方法:**

选择Masaoka-Koga分期Ⅰ期、Ⅱ期且术前没有接受新辅助治疗的患者，根据术中胸腺切除程度，分为胸腺切除组及胸腺瘤切除组。对比分析两组患者的临床特点及预后差异。

**结果:**

共有1, 047例患者纳入研究，其中胸腺切除组入组796例患者、胸腺瘤切除组入组251例患者。对于术前合并重症肌无力（myasthenia gravis, MG）的患者，胸腺切除组术后的MG的缓解率明显优于胸腺瘤切除组（91.6% *vs* 50.0%, *P* < 0.001）。胸腺切除组的10年总体生存率（overall survival, OS）为90.9%，胸腺瘤切除组的10年OS为89.4%，两者之间没有统计学差异（*P*=0.732）。胸腺切除组术后复发率为3.7%，胸腺瘤切除组术后复发率为6.2%，两组之间无统计学差异（*P*=0.149）。进一步分层分析显示，对于Masaoka-Koga Ⅰ期患者，胸腺切除组和胸腺瘤切除组在复发率上没有差异（3.2% *vs* 1.4%, *P*=0.259）；然而在Masaoka-Koga Ⅱ期患者中，胸腺切除组的复发率明显低于胸腺瘤切除组的复发率（2.9% *vs* 14.5%, *P*=0.001）。

**结论:**

胸腺切除是治疗胸腺上皮肿瘤的标准手术方式，特别是对于Masaoka-Koga Ⅱ期及合并MG的患者。

## Abstract (en)

**Background and objective:**

To evaluate the surgical outcomes of tumor resection with or without total thymectomy for thymic epithelial tumors (TETs) using the Chinese Alliance for Research in Thymomas (ChART) retrospective database.

**Methods:**

Patients without preoperative therapy, who underwent surgery for early-stage (Masaoka-Koga stage Ⅰ and Ⅱ) tumors, were enrolled for the study. They were divided into thymectomy and thymomectomy groups according to the resection extent of the thymus. Demographic and surgical outcomes were compared between the two patients groups.

**Results:**

A total of 1, 047 patients were enrolled, with 796 cases in the thymectomy group and 251 cases in the thymomectomy group. Improvement rate of myasthenia gravis (MG) was higher afer thymectomy than afer thymomectomy (91.6% *vs* 50.0%, *P* < 0.001). Ten-year overall survival was similar between the two groups (90.9% afer thymectomy and 89.4% afer thymomectomy, *P*=0.732). Overall, recurrence rate was 3.1% afer thymectomy and 5.4% afer thymomectomy, with no signifcant difference between the two groups (*P*=0.149). Stratifed analysis revealed no signifcant difference in recurrence rates in Masaoka-Koga stage Ⅰ tumors (3.2% *vs* 1.4%, *P*=0.259). However in patients with Masaoka-Koga stage Ⅱ tumors, recurrence was signifcantly less afer thymectomy group than afer thymomectomy (2.9% *vs* 14.5%, *P*=0.001).

**Conclusion:**

Tymectomy, instead of tumor resection alone, should still be recommended as the surgical standard for thymic malignancies, especially for stage Ⅱ tumors and those with concomitant MG.

手术切除是目前治疗胸腺上皮肿瘤的主要手段。由于所有的胸腺上皮肿瘤都具有一定的恶性生物学行为，且术中判断肿瘤包膜是否完整具有一定的困难，手术时应完整切除肿瘤及其周围的胸腺、脂肪组织而不是单纯将肿瘤剥离出去^[1]^。但对于胸腺切除的程度目前尚未达成共识^[2-4]^。虽然有研究证明胸腺切除可以降低术后新发重症肌无力（myasthenia gravis, MG）的几率以及局部复发率^[5, 6]^，但也有学者认为对于那些无外侵及无合并MG胸腺瘤患者，胸腺瘤切除也可以达到同样的治疗效果^[2, 3]^。随着目前计算机断层扫描（computed tomography, CT）筛查肺癌的开展，越来越多的早期的胸腺瘤也被检查出，且随着近年来胸腔镜技术的开展也使得越来越多的患者接受了胸腔镜下胸腺瘤切除术^[7]^。尽管有一些单中心研究证明胸腺切除和胸腺瘤切除治疗胸腺肿瘤在无病生存率上没有差异，但这些研究的随访时间相对较短^[2, 3]^。鉴于胸腺肿瘤发病率较低，生长缓慢，目前仍需要一个多中心、大样本量的研究证明手术切除范围对于胸腺肿瘤患者预后的影响。本研究拟利用中国胸腺肿瘤协作组（Chinese Alliance for Research in Thymomas, ChART）胸腺肿瘤回顾性数据库，通过对比Masaoka Ⅰ期/Ⅱ期肿瘤手术结果的差异，探讨胸腺切除范围的不同对早期胸腺肿瘤预后的影响。

## 1 材料和方法

ChART由中国18家三级甲等医院组成，共回顾性收集了自1994年-2012年共2, 104例胸腺肿瘤患者的资料。本研究共入组1047例没有接受术前新辅助治疗的早期胸腺肿瘤（Masaoka分期Ⅰ期、Ⅱ期）患者。临床资料的收集包括：患者一般资料、有无合并MG及其他自身免疫性疾病、手术情况、术后组织学分型、术后临床病理分期以及随访数据。采用美国重症肌无力基金会（Myasthenia Gravis Foundation of America, MGFA）分型及治疗后状况分类标准（MGFA post-intervention status）^[8]^对MG患者进行术前分型及手术疗效评价。采用世界卫生组织（World Health Organization, WHO）2004年分型标准进行组织学分型，采用Masaoka-Koga分期进行临床病理分期^[9]^。

手术径路包括经胸骨正中切口、经肋间切口以及胸腔镜径路，由于本研究为多中心回顾性研究，手术径路的选择没有统一标准，由手术医生根据自身习惯决定。同样的，术后辅助治疗也没有统一标准, 据医生对复发风险的主观评价进行。

根据胸腺切除范围，所有患者分为胸腺切除组以及胸腺瘤切除组。胸腺切除包括全胸腺切除及胸腺次全切除，共纳入796例患者，术中除完整切除肿瘤以外，还切除前纵隔脂肪在内的全部胸腺组织；胸腺瘤切除组共248例患者，切除范围包括完整切除肿瘤及周围胸腺或受累一侧的胸腺。

随访截止日期为2013年10月，本组患者平均随访时间38个月，随访率为78.4%。

统计数据SPSS 18.0 SPSS（Chicago, GA: SPSS）进行统计分析，采用*t*-检验、*χ*^2^检验对比两组患者特点，采用*Kaplan-Meier*法、*Log-rank*检验进行生存分析。*P*＜0.05为差异有统计学意义。

## 2 结果

纳入研究的1, 047例患者中，男性504例，女性543例，平均年龄（51.3±12.1）（15-83）岁。1, 033例（98.7%）患者接受了完整的手术切除（R0切除），14例（1.1%）患者有镜下残留（R1切除）。总共有310例（29.6%）患者接受了术后辅助治疗（包括术后辅助化疗或者辅助放疗）。入组患者的临床资料见表 1。两组患者的性别、年龄、肿瘤直径均无统计学差异。Masaoka Ⅰ期的患者在胸腺瘤切除组（70.9%）中的比例高于胸腺切除组（65.7%），但两者之间没有统计学差异（*P*=0.126）。然而胸腺癌在胸腺瘤切除组中的比例明显高于胸腺切除组（17.1 % *vs* 6.4%, *P*=0.007）。

在手术方式上，胸腺切除组主要采用经胸骨正中切口手术，而胸腺瘤切除组主要采用经肋间隙切口手术，两组手术方式有明显统计学差异（*P*＜0.001）。胸腔镜手术在两组中的比例相当。肿瘤完整切除（R0）的比例在两组患者中相似（*P*=0.267），但是胸腺瘤切除组的患者接受术后辅助治疗的比例要高于胸腺切除组（37.7% *vs* 28.5%, *P*=0.007）。

**1:** 胸腺切除组及胸腺瘤切除组患者的临床资料比较 Comparison of patient characteristics between thymectomy and thymomectomy

Variables	Thymectomy(*n*=796)	Thymomectomy(*n*=251)	*P*
Gender			0.371
Male	377(47.4%)	127 (50.6%)	
Female	419(52.6%)	124(49.4%)	
Age(yr, mean±SD)	50.9±12.2	52.3±11.9	0.628
Tumor size(cm, mean±SD)	6.67±2.9	6.68±3.4	0.902
Preoperative MG	247(31%)	15(6%)	＜0.001
Masaoka staging			0.126
Stage Ⅰ	523(65.7%)	178(70.9%)	
Stage Ⅱ	273(34.3%)	73(29.1%)	
WHO histological types			0.001
A+AB	348(43.7%)	100(39.8%)	
B1+B2+B3	397(49.9%)	108(43.1%)	
Carcinoid+Ca	51(6.4%)	43(17.1%)	
Resection state			0.267
R0	786(98.7%)	247(98.4%)	
R1	10(1.3%)	4(1.6%)	
Surgical approach			＜0.001
Sternotomy	498(62.6%)	23(9.2%)	
Thoracotomy	78(9.8%)	170(68%)	
VATS	220(27.6%)	57(22.8%)	
Adjuvant therapy			0.007
Surgery only	554(71.5%)	154(62.3%)	
Adjuvant therapy	217(28.5%)	93(37.7%)	
MG: myasthenia gravis; WHO: World Health Organization; SD: Standard Deviation; VATS: video-assisted thoracic surgery. 注：本表得到版权所有者© 2011-2016 Journal of Thoracic Disease复制许可。			

入组患者中，共有262例患者术前合并有MG。绝大部分患者（*n*=247, 94%）接受了胸腺切除术，只有15例（6%）患者接受了胸腺瘤切除。MG的缓解率在胸腺切除患者中的比例要明显高于胸腺瘤切除患者中的比例（91.6% *vs* 50.0%, *P*＜0.001）。胸腺切除组有2例（0.81%）患者术后新发MG。

胸腺切除组的10年总体生存率（overall survival, OS）为90.9%，胸腺瘤切除组的10年OS为89.4%，两者之间没有统计学差异（*P*=0.732，图 1）。根据Masaoka-Koga分期进行分层分析，结果显示Ⅰ期及Ⅱ期的患者在总体生存率上均无明显差异（图 2、图 3）。胸腺切除组的术后复发率为3.1%，胸腺瘤切除组的术后复发率为5.4%，两者之间没有统计学差异（*P*=0.149）；分层分析显示，对于Masaoka-Koga Ⅰ期的患者，胸腺瘤切除组的复发率和胸腺切除组相似（3.2% *vs* 1.4%, *P*=0.259）。而对于MasaokaKoga Ⅱ期的患者，胸腺瘤切除组的复发率要明显高于胸腺切除组（2.9% *vs* 14.5%, *P*=0.001）。

**1:**
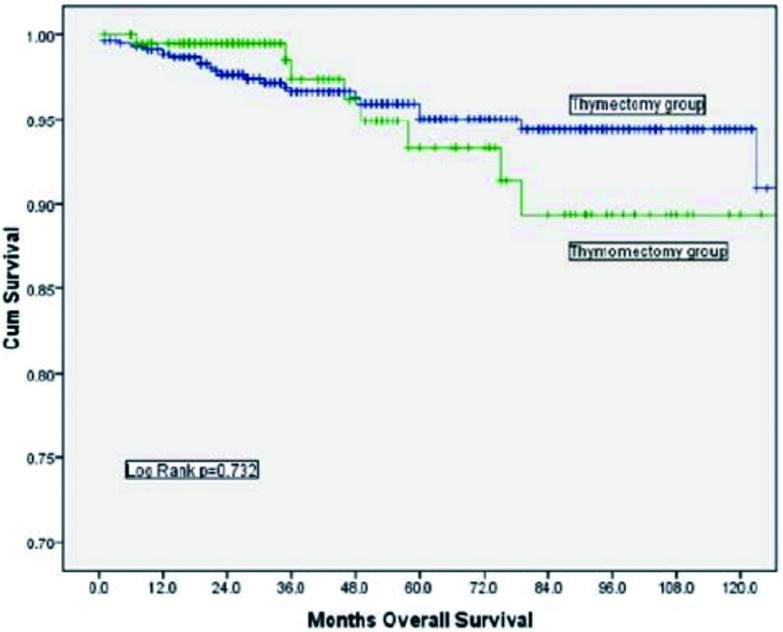
胸腺切除组与胸腺瘤切除组总体生存率比较（*P*=0.732） Comparison of overall survival between thymectomy and thymomectomy (*P*=0.732)

**2:**
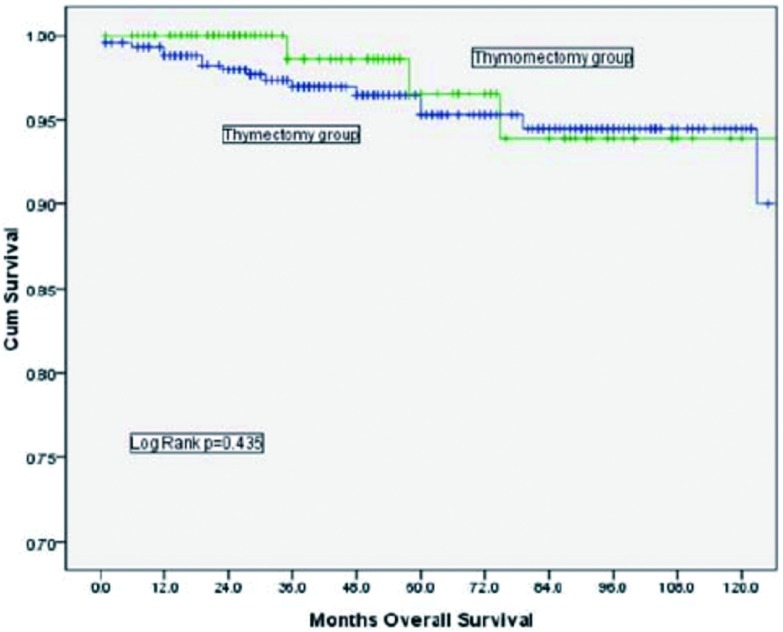
Masaoka-Koga Ⅰ期肿瘤患者，胸腺切除组与胸腺瘤切除组总体生存率比较（*P*=0.435） Comparison of overall survival between thymectomy and thymomectomy among patients with Masaoka-Koga stage Ⅰ tumors (*P*=0.435)

**3:**
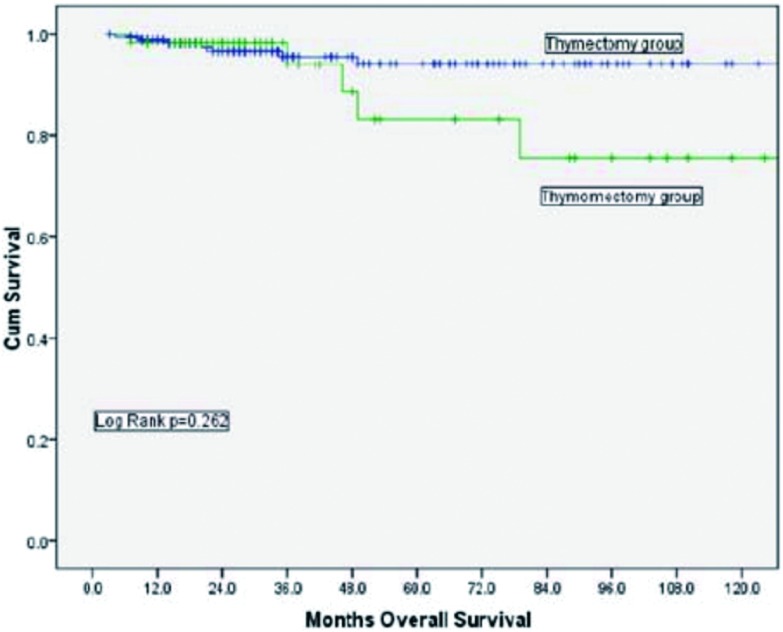
Masaoka-Koga Ⅱ期肿瘤患者，胸腺切除组与胸腺瘤切除组总体生存率比较（*P*=0.262） Comparison of overall survival between thymectomy and thymomectomy among patients with Masaoka-Koga stage Ⅱ tumors (*P*=0.262)

## 3 讨论

经胸骨正中切口行胸腺切除术是治疗胸腺上皮肿瘤的金标准，特别是当患者术前合并MG等自身免疫性疾病时，更需要在行胸腺切除的同时，清扫两侧纵隔脂肪^[1]^。但是考虑到经胸骨正中切口手术创伤较大，且有1%-5%的比例术后发生纵隔感染的风险^[4]^。为减小手术损伤，许多医生选择经肋间隙、颈部以及电视辅助胸腔镜手术（videoassisted thoracic surgery, VATS）等切口手术^[5-7]^。对于早期胸腺肿瘤，这些切口可以完成胸腺肿瘤的完整切除，但是通过肋间隙切口行全胸腺切除则技术上相对比较困难。随着影像及外科技术的进展，越来越多的直径相对较小的胸腺瘤在偶然体检发现，并且相当一部分患者接受了胸腔镜手术。VATS下行胸腺切除增加了无名静脉出血的风险，因此部分医生选择行胸腺瘤切除术^[3]^。因此，我们有必要分析手术切除的范围对早期胸腺源性肿瘤预后的影响。

胸腺上皮肿瘤的预后与肿瘤分期、WHO分型、手术切除程度以及术后辅助治疗有关^[10]^。早期胸腺肿瘤预后良好，术后复发率、死亡率较低^[11]^。目前的研究显示，Masaoka-Koga Ⅰ期和Ⅱ期胸腺肿瘤10年的总体生存率高达90%以上。几项关于胸腺切除范围对预后影响的研究均显示，胸腺瘤切除患者的术后复发率、生存率与胸腺切除患者无统计学差异^[2-4]^。本组研究结果显示，10年的总体生存率在胸腺切除组和胸腺瘤切除组相似（90.9% *vs* 89.4%, *P*=0.732）。然而这并不意味着胸腺切除和胸腺瘤切除在肿瘤治疗上相似。在胸腺瘤切除组中，有更多的患者接受了术后辅助治疗；另一方面，胸腺瘤是一种相对比较“惰性”的肿瘤，生长缓慢，即使在肿瘤复发后患者仍可以长期生存。鉴于这些原因，我们认为用复发率可以更好的评价手术切除的范围对于早期胸腺瘤患者生存的影响。在本组研究中，我们发现虽然两组患者的复发率相似（3.1% *vs* 5.4%, *P*=0.149），但是进一步的分层分析显示，对于Masaoka-Koga Ⅱ期患者，胸腺瘤切除组的复发率要明显高于胸腺切除组（2.9% *vs* 14.5%, *P*=0.001）。这是因为对于具有完整包膜的Masaoka-Koga Ⅰ期患者而言，无论是胸腺切除或者胸腺瘤切除，完整的切除肿块可能即达到肿瘤学上的根治效果。但对于Masaoka-Koga Ⅱ期的患者，由于术中无法准确判断肿瘤外侵的程度及肿瘤边缘的位置，所以在行胸腺瘤切除时有可能会造成肿瘤的意外残留，这也解释了患者胸腺瘤切除对于Masaoka-Koga Ⅱ期的胸腺瘤患者具有相对较高的复发率的原因。

理论上，无论胸腺切除或者胸腺瘤切除对MasaokaKoga Ⅰ期都可以达到根治效果，因为Ⅰ期的胸腺肿瘤包膜完整而没有外侵。在本组研究中，Masaoka-Koga Ⅰ期患者的复发率在两组患者中并无统计学差异。但是，通过术前的检查或者术中准确判断肿瘤处于Masaoka-Koga Ⅰ期尚有困难。CT是目前应用最广泛的胸腺肿瘤的影像学检查。国际胸腺恶性肿瘤兴趣小组也推荐将CT作为术前分期的标准检查^[12]^。然而，很少有研究证明其准确性及实用性^[13]^。总体来说，CT对于判断早期胸腺上皮肿瘤的敏感性和特异性都不是很高，更无法鉴别Masaoka-Koga Ⅰ期和Ⅱ期的肿瘤^[14]^。虽然正电子发射型计算机断层显像（positron emission computed tomography, PET）-CT对于胸腺瘤和胸腺癌有一定的鉴别价值，但对于那些没有明显外侵或者直径较小的肿瘤，PET-CT则无法准确的判断其病理分期^[15]^。和处理其他恶性肿瘤相似，手术治疗的目的一是为了完整切除肿块，二是为了更准确的病理分期。所以对于临床上判断Ⅰ期的胸腺肿瘤，我们也推荐进行完整的胸腺切除。

对于术前合并MG的胸腺上皮肿瘤患者，胸腺切除的手术效果已得到证实，有效率可以达到73%-89%，完全缓解率可以达到28%-52%^[8, 15-18]^。目前研究多比较胸腺切除术与胆碱酯酶抑制剂、免疫抑制剂等内科治疗的效果，显示胸腺切除术后患者的缓解率高于内科治疗患者^[16, 17]^, 但尚未有研究探讨胸腺切除范围对此的影响。在本组研究中，有15例术前合并MG的患者行胸腺瘤切除，术后MG缓解率为27.3%，远低于胸腺切除组MG患者术后缓解率，这个和Sonet等^[19]^报道的相一致。这也证明对于术前合并MG的患者，胸腺切除加纵隔脂肪的清扫将有助于提高重症肌无力的缓解率。我们建议对于合并重症肌无力的胸腺瘤患者进行胸腺切除来保证治疗效果。

对于术前不合并MG的患者，胸腺肿瘤切除术后仍有发生MG的风险，文献^[5, 6]^报道其比例大致为1.5%-28%。胸腺切除范围对术后MG的发生有无影响，目前尚不清楚。Ito等^[18]^报道，胸腺切除组术后MG发生率为5%，胸腺瘤切除组术后MG发生率为4.2%，两组比较无统计学差异，Onuki^[2]^、Tseng^[3]^等报道的结果与此类似。本组数据显示：术前不伴MG的患者，术后仅胸腺切除组有2例（0.81%）患者新发MG。这一结果说明术后新发MG是非常少见的。全胸腺切除并不能减少术后新发MG的发生率。

本研究尚有诸多局限之处：①本组研究为多中心回顾性研究存在选择偏倚，比如全胸腺切除组Ⅰ期患者比例大于Ⅱ期患者；②胸腺切除范围、术后辅助治疗方式多由医生根据习惯选择，缺乏统一标准；③平均随访时间仅有50个月，失访率较高；尽管我们进行了分层分析来尽可能避免混杂便宜，但更高级别的证据还有待于多中心前瞻性随机对照研究及长时间随访观察的结果。

## 4 结论

本组研究结果显示，尽管胸腺切除和胸腺瘤切除在总体生存率上相似，目前尚无足够的证据推荐对于早期的胸腺肿瘤患者仅进行胸腺肿瘤切除。鉴于目前临床上于术前尚无法准确判断病理Masaoka-Koga Ⅰ期的胸腺肿瘤，且Ⅱ期的胸腺肿瘤复发率在胸腺瘤切除中要明显高于胸腺切除，我们建议常规进行胸腺切除来保证手术的根治效果及更准确的病理分期，特别是对于那些合并重症肌无力的患者。

## References

[1] Alper T, Joshua S, Marcin Z (2011). Standard terms, defnitions, and policies for minimally invasive resection of thymoma. J Thorac Oncol.

[2] Onuki T, Ishikawa S, Iguchi K (2009). Limited thymectomy for stage Ⅰ or Ⅱthymomas. Lung cancer.

[3] Yen-Chiang T, Chih-Cheng H, Hsin-Yi H (2013). Is thymectomy necessary innonmyasthenic patients with early thymoma?. J Thorac Oncol.

[4] Sakamaki Y, Kido T, Yasukawa M (2008). Alternative choices of total and partial thymectomy in video-assisted resection of noninvasive thymomas. Surg Endosc.

[5] Nakajima J, Murakawa T, Fukami T (2008). Postthymectomy myasthenia gravis:relationship with thymoma and antiacetylcholine receptor antibody. Ann Thorac Surg.

[6] Kazuya K, Yasumasa M (2005). Myasthenia gravis appearing afer thymectomy for thymoma. Eur J Cardiothorac Surg.

[7] Pennathur A, Qureshi I, Schuchert MJ (2011). Comparison of surgical techniques for early-stage thymoma:Feasibility of minimally invasive thymectomy and comparison with open resection. J Thorac Cardiovasc Surg.

[8] Jaretzki A, Barohn R, Ernstoff R (2000). Myasthenia gravis Recommendations for clinical research standards. Neurology.

[9] Masaoka A, Monden Y, Nakahara K (1981). Follow‐up study of thymomas with special reference to their clinical stages. Cancer.

[10] Wright CD, Wain JC, Wong DR (2005). Predictors of recurrence in thymic tumors:Importance of invasion, World Health Organization histology, and size. J Thorac Cardiovasc Surg.

[11] Kondo K, Monden Y (2003). Therapy for thymic epithelial tumors:a clinical study of 1, 320 patients from Japan. Ann Thorac Surg.

[12] Marom EM, Rosado-de-Christenson ML, Bruzzi JF (2011). Standard report terms for chest computed tomography reports of anterior mediastinal masses suspicious for thymoma. J Thorac Oncol.

[13] Marom EM, Milito MA, Moran CA (2011). Computed tomography findings predicting invasiveness of thymoma. J Thorac Oncol.

[14] Priola AM, Priola SM, Di Franco M (2010). Computed tomography and thymoma:distinctive fndings in invasive and noninvasive thymoma and predictive features of recurrence. Radiol Med.

[15] Benveniste MF, Moran CA, Mawlawi O (2013). FDG PET-CT aids in the preoperative assessment of patients with newly diagnosed thymic epithelial malignancies. J Thorac Oncol.

[16] Roth T, Ackermann R, Stein R (2002). Tirteen years follow-up afer radical transsternal thymectomy for myasthenia gravis.Do short-term results predict long-term outcome?. Eur J Cardiothorac Surg.

[17] Buckingham JM, Howard Jr FM, Bernatz PE (1976). The value of thymectomy in myasthenia gravis:a computer-assisted matched study. Anna Surg.

[18] Ito M, Fujimura S, Monden Y (1992). A retrospective group study on postthymectomy myasthenia gravis. Nihon Kyobu Geka Gakkai Zasshi.

[19] Sonett JR, Jaretzki III A (2008). Thymectomy for nonthymomatous myasthenia gravis. Ann N Y Acad Sci.

